# Serum Uric Acid and Cardiovascular or All-Cause Mortality in Peritoneal Dialysis Patients: A Systematic Review and Meta-Analysis

**DOI:** 10.3389/fcvm.2021.751182

**Published:** 2021-11-03

**Authors:** Zhi-qiang Liu, Zhi-wen Huang, Shu-ling Kang, Chan-chan Hu, Fa Chen, Fei He, Zheng Lin, Feng Yang, Zhi-jian Hu

**Affiliations:** ^1^Department of Epidemiology and Health Statistics, School of Public Health, Fujian Medical University, Fuzhou, China; ^2^Fuzhou Center for Disease Control and Prevention, Fuzhou, China; ^3^Department of Preventive Medicine, School of Public Health, Fujian Medical University, Fuzhou, China; ^4^Department of Nephrology, Affiliated Fuzhou First Hospital of Fujian Medical University, Fuzhou, China

**Keywords:** serum uric acid, cardiovascular mortality, all-cause mortality, peritoneal dialysis, meta-analysis

## Abstract

**Background:** Studies have shown inconsistent associations between serum uric acid (SUA) levels and mortality in peritoneal dialysis (PD) patients. We conducted this meta-analysis to determine whether SUA levels were associated with cardiovascular or all-cause mortality in PD patients.

**Methods:** PubMed, Embase, Web of Science, the Cochrane Library, CNKI, VIP, Wanfang Database, and trial registry databases were systematically searched up to April 11, 2021. Cohort studies of SUA levels and cardiovascular or all-cause mortality in PD patients were obtained. Random effect models were used to calculate the pooled adjusted hazard ratio (*HR*) and corresponding 95% confidence interval (*CI*). Sensitivity analyses were conducted to assess the robustness of the pooled results. Subgroup analyses and meta-regression analyses were performed to explore the sources of heterogeneity. Funnel plots, Begg's tests, and Egger's tests were conducted to evaluate potential publication bias. The GRADE approach was used to rate the certainty of evidence. This study was registered with PROSPERO, CRD42021268739.

**Results:** Seven studies covering 18,113 PD patients were included. Compared with the middle SUA levels, high SUA levels increased the risk of all-cause mortality (*HR* = 1.74, 95%*CI*: 1.26–2.40, *I*^2^ = 34.8%, τ^2^ = 0.03), low SUA levels were not statistically significant with the risk of all-cause or cardiovascular mortality (*HR* = 1.04, 95%*CI*: 0.84–1.29, *I*^2^ = 43.8%, τ^2^ = 0.03; *HR* = 0.89, 95%*CI*: 0.65–1.23, *I*^2^ = 36.3%, τ^2^ = 0.04; respectively). Compared with the low SUA levels, high SUA levels were not statistically associated with an increased risk of all-cause or cardiovascular mortality (*HR* = 1.19, 95%*CI*: 0.59–2.40, *I*^2^ = 88.2%, τ^2^ = 0.44; *HR* = 1.22, 95%*CI*: 0.39–3.85, *I*^2^ = 89.3%, τ^2^ = 0.92; respectively).

**Conclusion:** Compared with middle SUA levels, high SUA levels are associated with an increased risk of all-cause mortality in PD patients. SUA levels may not be associated with cardiovascular mortality. More high-level studies, especially randomized controlled trials, are needed to determine the association between SUA levels and cardiovascular or all-cause mortality in PD patients.

**Systematic Review Registration:** https://www.crd.york.ac.uk/prospero/display_record.php?ID=CRD42021268739, identifier: CRD42021268739.

## Introduction

Chronic kidney disease (CKD) has become one of the most critical public health problems globally. The global prevalence of CKD is estimated at 13.4%, which is likely to rise further as the population ages and diabetes increases (1). In 2017, about 1.2 million people died of CKD worldwide, with a 41.5% increase in global mortality between 1990 and 2017 (2). End-stage renal disease (ESRD) is a critical aspect of the severe burden of CKD. The treatment of ESRD includes supportive care and renal replacement therapy, including hemodialysis (HD), peritoneal dialysis (PD), and renal transplantation (3). Liyanage et al. (4) reported ESRD renal replacement therapy data in 123 countries or regions, and the results showed that about 2.62 million patients received renal replacement therapy in 2010, including about 2.05 million dialysis patients. In the world, PD is not as extensive as HD. In a survey of 125 countries, PD and HD can be implemented in 95 (76%) and 119 (95%) countries, respectively (5). However, compared with HD, PD has characteristics of the lower cost, the convenience of home therapy, and a flexible schedule (6). Recent studies have consistently shown that PD patients have higher early survival than HD patients (7–9).

Uric acid is the ultimate oxidation product of purine catabolism in humans, including exogenous and endogenous purine metabolism (10, 11). Hyperuricemia is one of the risk factors for kidney disease, diabetes, and hypertension (12, 13). High uric acid levels can also lead to increased all-cause mortality of obesity (14) and chronic heart failure (15). Two-thirds of uric acid is excreted in the urine (16). Uric acid levels are often elevated in patients with CKD due to decreased renal function. Meta-analyses of CKD patients have shown a U-shaped trend between SUA and all-cause mortality (17), and higher SUA levels are significantly associated with increased risk of cardiovascular mortality (18). Another meta-analysis of HD patients has shown that high SUA levels are a protective factor for cardiovascular mortality (19). However, there is no meta-analysis about the association between SUA levels and mortality in PD patients.

The association between SUA levels and all-cause mortality in PD patients is inconsistent. Feng et al. (20) found that high SUA was associated with increased all-cause mortality; Lai et al. (21) found that high SUA was associated with low risk of all-cause mortality; while Sugano et al. (22) found a U-shaped relationship between SUA levels and all-cause mortality. Similarly, the association between SUA levels and cardiovascular mortality in PD patients is controversial. Lai et al. (21) found that high SUA levels were associated with low risk of cardiovascular mortality; Xia et al. (23) found that the higher the SUA levels, the higher the cardiovascular mortality; while Xiang et al. (24) found that there was no significant relationship between SUA levels and cardiovascular mortality. Therefore, we performed a systematic review and meta-analysis to summarize the relevant studies and clarify the association between SUA levels and cardiovascular or all-cause mortality in PD patients. The objective of the meta-analysis can be described in the form of a PI(E)CO statement as follows: in peritoneal dialysis patients (population), how do different baseline serum uric acid levels (low vs. middle, high vs. middle, high vs. low) (exposure vs. comparator) influence the cardiovascular or all-cause mortality (outcomes)?

## Materials and Methods

This study was conducted following the Preferred Reporting Items for Systematic reviews and Meta-Analyses (PRISMA) statement (25). The protocol was registered at PROSPERO (CRD42021268739, https://www.crd.york.ac.uk/prospero/display_record.php?ID=CRD42021268739).

### Literature Search

We systematically searched PubMed, Embase, Web of Science, the Cochrane Library, Chinese National Knowledge Infrastructure (CNKI), Chinese Scientific Journal Database (VIP), and Wanfang Database to identify relevant studies up to April 11, 2021. We used the following search strategies to retrieve articles: (“uric acid” OR hyperuricemia OR urate) AND (peritoneal dialysis OR dialysis) AND (mortality OR death OR survival OR prognosis OR outcome OR cardiovascular disease OR cardiovascular events OR stroke). Besides, we searched the clinical trial registries (https://www.clinicaltrials.gov/) using the keywords of “uric acid” and “peritoneal dialysis” to identify the relevant unpublished studies. We also manually checked the reference lists of retrieved studies to identify potentially missing relevant studies.

### Inclusion and Exclusion Criteria

Inclusion criteria: (1) cohort study design; (2) patients with chronic kidney disease receiving peritoneal dialysis (which uses the peritoneum as a dialysis membrane by means of dispersion and ultrafiltration) (26); (3) available baseline data of SUA levels; (4) determining the effect of baseline SUA on prognosis, with an effect size of *HR*; (5) outcome included cardiovascular or all-cause mortality.

Exclusion criteria: (1) letters, animal studies, reviews, conference reports, meta-analyses; (2) when the same population appeared in different studies, only the one with the longest follow-up period or the most information was included.

SUA levels were used as a categorical variable divided into low, middle, and high levels. The classification of SUA levels was based on the definition in each original study. The lowest uric acid group was defined as low level, the highest uric acid group was defined as high level, and the middle uric acid group was defined as middle level.

### Data Extraction

Two reviewers independently screened each record and extracted data from eligible studies, and any discrepancies were resolved by a comprehensive discussion and reviewed by a third investigator. Extraction of information included the first author, publication year, country, sample size, age, male proportion, the proportions of hypertension and diabetes, baseline uric acid, follow-up period, all-cause and cardiovascular death, comparison, adjusted *HR* and 95%*CI* (the most fully adjusted), and adjusted factors.

### Quality Assessment

The Newcastle-Ottawa Scale (NOS) criteria for cohort studies were used to assess the quality of each included study. The following eight items were evaluated: (1) representativeness of the exposed cohort; (2) selection of the non-exposed cohort; (3) ascertainment of exposure; (4) demonstration that outcome of interest was not present at start of study; (5) comparability of cohorts on the basis of the design or analysis; (6) assessment of outcome; (7) was follow-up long enough for outcomes to occur; (8) adequacy of follow up of cohorts. Selection, comparability, and outcome are three aspects of the NOS criteria. Nine points are the highest score, and the overall quality scores can be divided into good (7–9 points), average (4–6 points), and poor (0–3 points).

The rating of the overall quality of the evidence was undertaken using the Grades of Recommendation, Assessment, Development, and Evaluation (GRADE) approach applied to prognostic studies (27).

The appraisals were processed independently by two reviewers, and the final results were obtained by consensus.

### Statistical Analysis

We used Stata 15.0 (Stata Corp, College Station, TX, USA) for statistical analysis, and all tests were two-tailed. We calculated the significance of the pooled *HR* by *Z*-test, and *P* < 0.05 was considered statistically significant. We used the *Q*-test, *I*^2^ statistics, and *Tau-squared* (τ^2^) to judge the magnitude of heterogeneity among studies. We applied the random-effects model to calculate pooled *HR* and further used the meta-regression to explore the sources of heterogeneity. Subgroup analyses were performed by male proportion, sample size, hypertension proportion, and diabetes mellitus proportion. Sensitivity analyses were conducted by omitting each study to assess the robustness of the pooled results. The potential publication bias was qualitatively evaluated by funnel plots and further quantitatively evaluated by Begg's test (28) and Egger's test (29). If publication bias was present, results were adjusted using the trim and fill method (30).

## Results

### Summary of the Included Studies

**Figure 1:**
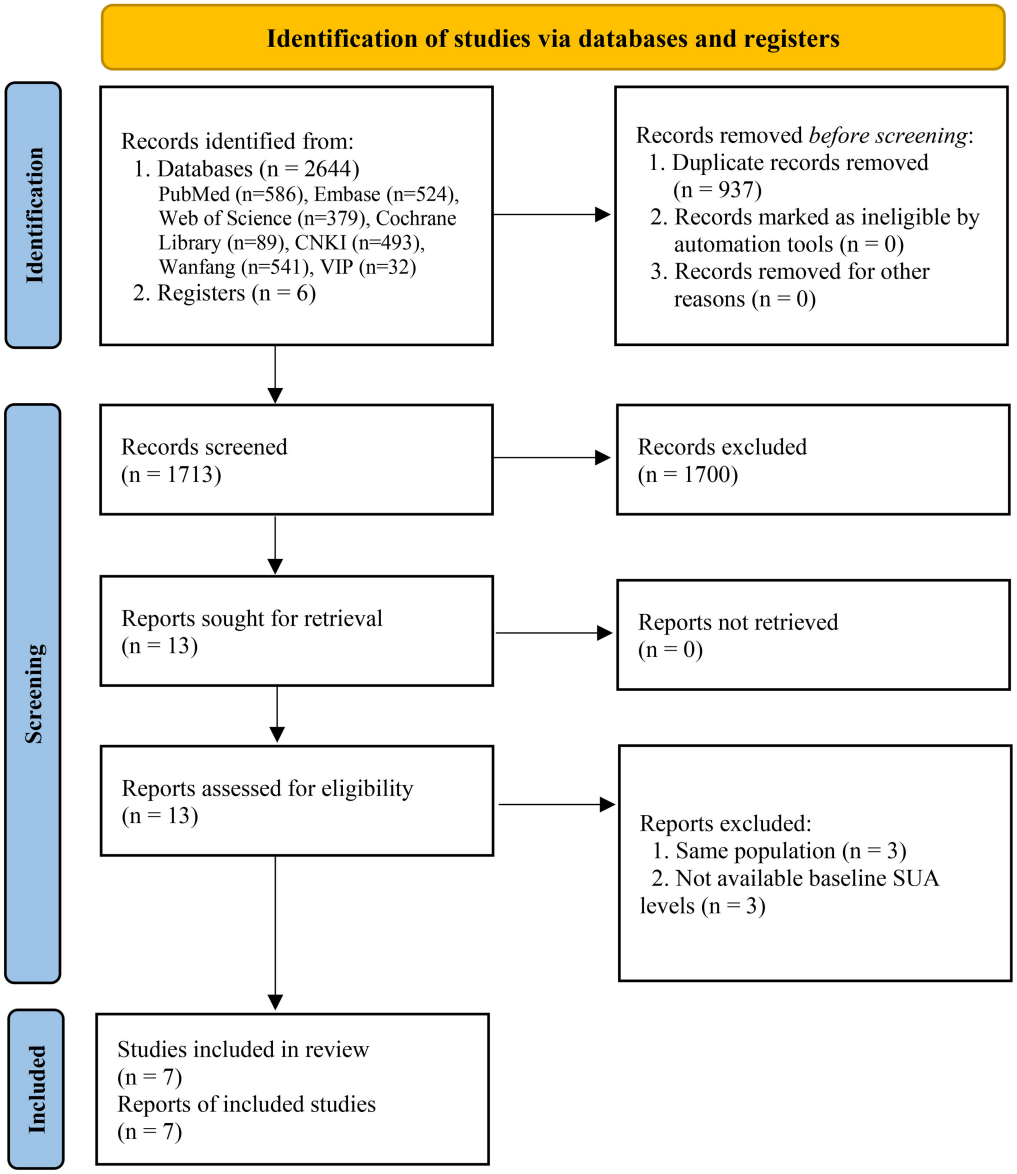
Flow diagram of systematic literature search.

Two thousand six hundred forty-four and 6 records were originally identified through databases and registers searching. After removing duplicates, 1,713 records were further reviewed. Based on the eligibility criteria, 1,700 records were excluded after screening titles and abstracts, 13 full-text articles were further assessed for eligibility, 6 articles were excluded with reasons. Finally, 7 eligible studies were included in the meta-analysis (20–24, 31, 32), including 7 for all-cause mortality (20–24, 31, 32) and 4 for cardiovascular mortality (21, 23, 24, 31) (Figure 1).

The included studies encompassed 18,113 patients with all-cause mortality data and 13,075 patients with cardiovascular mortality data. All seven studies reported sample size, mean age, baseline SUA, and follow-up period. The sample size of studies ranged from 140 to 9,405. The proportion of males, hypertension, and diabetes ranged from 45 to 63%, 65.7 to 94.3%, and 12.2 to 37.9%, respectively. All studies provided adjusted *HR* (95%*CI*) and adjusted factors (Table 1).

**Table 1:** Characteristics of 7 studies included in the meta-analysis.

**Author**	**Country**	**Patients (*n*)**	**Age (years)**	**Male (%)**	**Hypertension (%)**	**Diabetes (%)**	**Baseline uric acid (mg/dL)**	**Follow-up (months)**	**All-cause death (*n*)**	**CV death (*n*)**	**Comparison (mg/dL)**	**Adjusted *HR* (95%*CI*)**	**Adjustments**
Feng et al. (20)	China	156	54.1 ± 17.4	63.0	92.0	25.0	8.50 ± 2.10	31.3 ± 17.5	41	NA	Low vs. Middle ≤ 7.0 vs. 7.0–10.0	1.15 (0.20–6.61) All-cause mortality	Age, albumin, DM, hypertensive nephropathy, RRF.
											High vs. Middle ≥10.0 vs. 7.0–10.0	2.96 (1.29–6.79) All-cause mortality	
Dong et al. (31)	China	2,193	58.1 ± 15.5	49.1	NA	37.9	6.41 ± 1.87	Median 26.5	586	231	Low vs. Middle M: 2.09-5.79 vs. 5.80-7.38 FM: 1.74–5.37 vs. 5.38–6.65	0.81 (0.59–1.11) All-cause mortality 0.78 (0.45–1.35) CV mortality	Age, RRF, serum albumin, hemoglobin, phosphate, CRP, the history of CVD, diabetes, BMI, mean arterial pressure, LDLC, center size, gender.
											High vs. Low M: 7.39–16.7 vs. 2.09-5.79 FM: 6.66–8.08 vs. 1.74–5.37	1.21 (0.85–1.72) All-cause mortality 1.35 (0.74–2.46) CV mortality	
Xia et al. (23)	China	985	48.3 ± 15.4	58.4	65.7	23.0	7.00 ± 1.30	Median 25.3	144	64	Low vs. Middle M: <6.67 vs. 6.67–7.56 FM: <6.19 vs. 6.19–7.13	0.78 (0.50–1.22) All-cause mortality 0.51 (0.25–1.04) CV mortality	Age, BMI, Davies comorbidity score, hemoglobin, serum albumin, serum creatinine, albumin-corrected calcium, serum phosphorus, total triglyceride,
											High vs. Low M: 7.56 vs. <6.67 FM: >7.13 vs. <6.19	1.93 (1.27–2.93) All-cause mortality 3.31 (1.70–6.44) CV mortality	LDLC, residual kidney function, log-transformed high-sensitivity C-creative protein level, total Kt/V, use of allopurinol, loop diuretics, angiotensin-converting enzyme inhibitor, or angiotensin receptor blocker.
Lai et al. (21)	China	492	53.5 ± 15.3	48.0	85.2	34.6	7.20 (IQR, 6.30–8.20)	Median 36.4	127	74	Low vs. Middle M: ≤ 6.8 vs. 6.9–8.0 FM: ≤ 6.5 vs. 6.6–7.6	0.94 (0.63–1.40) All-cause mortality 0.96 (0.56–1.65) CV mortality	Age, sex, BMI, the pre-dialysis status, smoking status, medications (ACE inhibitor/ARB, erythropoiesis stimulating agents, furosemide, vitamin D, statin, allopurinol,
											High vs. Low M: ≥ 8.1 vs. ≤ 6.8 FM: ≥ 7.7 vs. ≤ 6.5	0.40 (0.24–0.67) All-cause mortality 0.40 (0.20–0.80) CV mortality	Calcium channel blocker); comorbidities (DM, hypertension, CVD, Charlson score); PD-related parameters (weekly total Kt/V urea, nPNA, dialysate-to-plasma (D/P) creatinine at 4 h, ultrafiltration, 24-h urine output, RRF), laboratory data (BUN, creatinine, albumin, GPT, WBC counts, alkaline phosphate, hemoglobin, ferritin, transferrin saturation, cholesterol, triglyceride, PTH, calcium, phosphate).
Xiang et al. (24)	China	9,405	52.5 ± 14.6	54.9	NA	12.2	7.07 ± 1.25	Median 29.4	1,226	515	Low vs. Middle <6.06 vs. 6.68–7.27	1.16 (0.95–1.43) All-cause mortality 1.17 (0.82–1.66) CV mortality	Age, sex, BMI, DM, CVD, RRF, hemoglobin, serum albumin, serum potassium, serum natrium, serum phosphorus, serum calcium, serum parathyroid hormone, serum creatinine, and fasting plasma glucose.
											High vs. Middle ≥8.04 vs. 6.68–7.27	1.48 (1.19–1.85) All-cause mortality	
Qiu et al. (32)	China	140	57.0 (44.0~65.0)	45.0	94.3	37.9	7.57 ± 2.46	Median 31.9	48	NA	Low vs. Middle <6.51 vs. 6.52–8.73	1.04 (0.46–2.36) All-cause mortality	Age, DM, hypertension, CVD, kalium, RRF, uric acid reduction medicine, diuretics, ESA, BMI.
											High vs. Low ≥8.73 vs. <6.51	2.31 (1.06–5.02) All-cause mortality	
Sugano et al. (22)	Japan	4,742	63.0 ± 14.0	61.5	NA	29.1	6.49 ± 1.40	Median 28.0	379	129	Low vs. Middle <5.0 vs. 7.0–7.5	1.80 (1.13–2.87) All-cause mortality	Age, sex, dialysis duration, BMI, UV, underlying disease, comorbid disease, medication and laboratory data.
											High vs. Middle >8.5 vs. 7.0–7.5	1.93 (1.15–3.24) All-cause mortality	

*Conversion factors for units: to convert uric acid in mg/dL to μmol/L, multiply by 59.48*.

*The group behind vs. is the reference group*.

*HR, hazard ratio; CI, confidence interval; M, male; FM, female; CRP, C-creative protein; BMI, body mass index; CV, cardiovascular; CVD, cardiovascular disease; DM, diabetes mellitus; LDLC, low-density lipoprotein cholesterol; NA, not available; IQR, interquartile range; PD, peritoneal dialysis; UV, urinary volume; RRF, residual renal function; ACE, angiotensin-converting enzyme; ARB, angiotensin II receptor blocker; nPNA, normalized protein nitrogen appearance; BUN, blood urea nitrogen; GPT, glutamic-pyruvic transaminase; WBC, white blood cell; PTH, intact parathyroid hormone; ESA, erythropoiesis stimulating agents*.

### Quality Assessment

All original studies included in this meta-analysis were cohort study designs, and their quality was assessed according to the NOS criteria. All studies were evaluated seven scores or above and rated as good. The details of the NOS scores were summarized in Table 2.

**Table 2:** Newcastle-Ottawa Scale of included studies.

**Study**	**Selection**				**Comparability**		**Outcome**			**Total**
	**(1)**	**(2)**	**(3)**	**(4)**	**(5A)**	**(5B)**	**(6)**	**(7)**	**(8)**	
Feng et al. (20)	1	1	1	0	1	1	1	1	0	7
Dong et al. (31)	1	1	1	0	1	1	1	1	0	7
Xia et al. (23)	1	1	1	1	1	1	1	1	0	8
Lai et al. (21)	1	1	1	0	1	1	1	1	0	7
Xiang et al. (24)	1	1	1	0	1	1	1	1	0	7
Qiu et al. (32)	1	1	1	0	1	1	1	1	0	7
Sugano et al. (22)	1	1	1	0	1	1	1	1	0	7

*(1) Representativeness of the exposed cohort; (2) Selection of the non-exposed cohort; (3) Ascertainment of exposure; (4) Demonstration that outcome of interest was not present at start of study; (5) Comparability of cohorts on the basis of the design or analysis: (5A) control age (or other important factors); (5B) control other factors; (6) Assessment of outcome; (7) Was follow-up long enough for outcomes to occur; (8) Adequacy of follow up of cohorts*.

### Serum Uric Acid Levels and All-Cause Mortality

**Figure 2:**
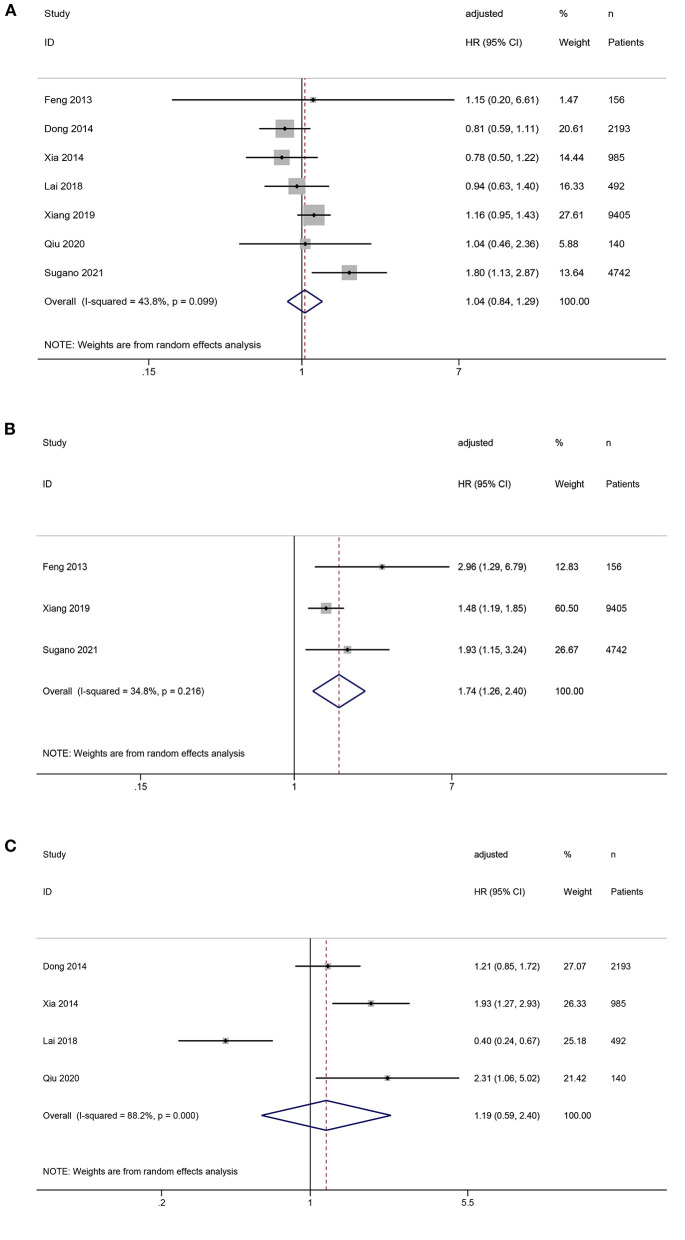
Forest plot of the association between serum uric acid levels and all-cause mortality in PD patients. **(A)** low vs. middle; **(B)** high vs. middle; **(C)** high vs. low.

All seven studies reported the association between SUA levels and all-cause mortality in PD patients. The pooled results showed that compared with middle SUA levels, low SUA levels were not statistically significant in the increased risk of all-cause mortality (*HR* = 1.04, 95%*CI*: 0.84–1.29, *I*^2^ = 43.8%, τ^2^ = 0.03), but the high SUA levels increased the risk of all-cause mortality (*HR* = 1.74, 95%*CI*: 1.26–2.40, *I*^2^ = 34.8%, τ^2^ = 0.03). Compared with low SUA levels, high SUA levels were not statistically significant with an increased risk of all-cause mortality (*HR* = 1.19, 95%*CI*: 0.59–2.40, *I*^2^ = 88.2%, τ^2^ = 0.44) (Figures 2A–C).

### Serum Uric Acid Levels and Cardiovascular Mortality

**Figure 3:**
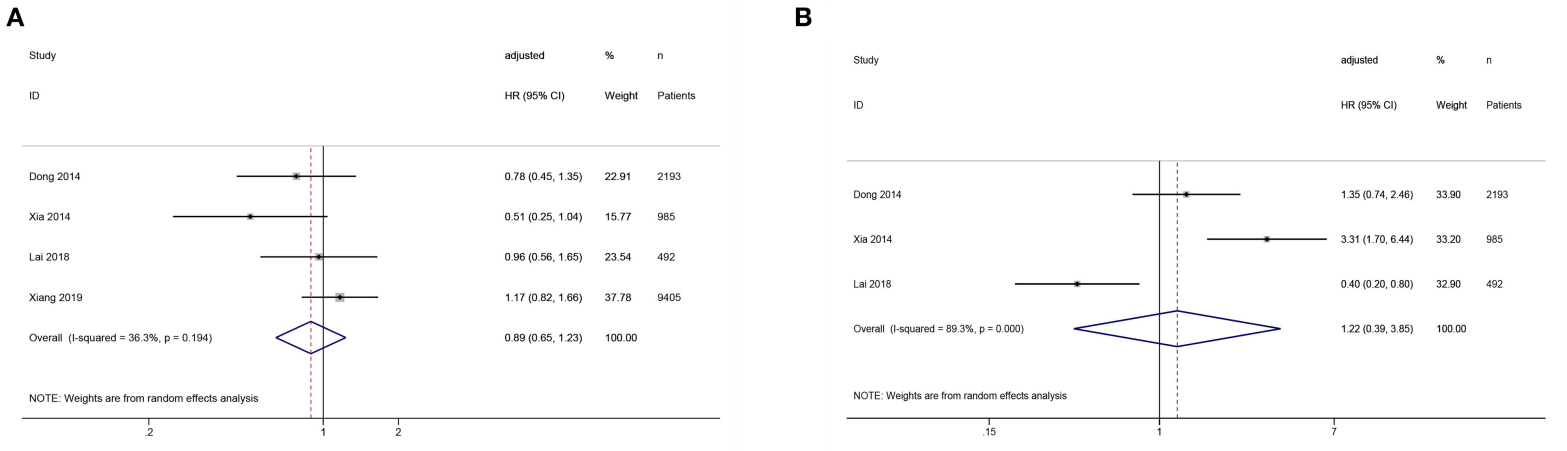
Forest plot of the association between serum uric acid levels and cardiovascular mortality in PD patients. **(A)** low vs. middle; **(B)** high vs. low.

Four studies reported the association between SUA levels and cardiovascular mortality in PD patients. Compared with middle SUA levels, low SUA levels were not associated with a statistically significant risk of cardiovascular mortality (*HR* = 0.89, 95%*CI*: 0.65–1.23, *I*^2^ = 36.3%, τ^2^ = 0.04). Compared with the low SUA levels, high SUA levels were not statistically associated with an increased risk of cardiovascular mortality (*HR* = 1.22, 95%*CI*: 0.39–3.85, *I*^2^ = 89.3%, τ^2^ = 0.92) (Figures 3A,B).

### Sensitivity Analysis

**Figure 4:**
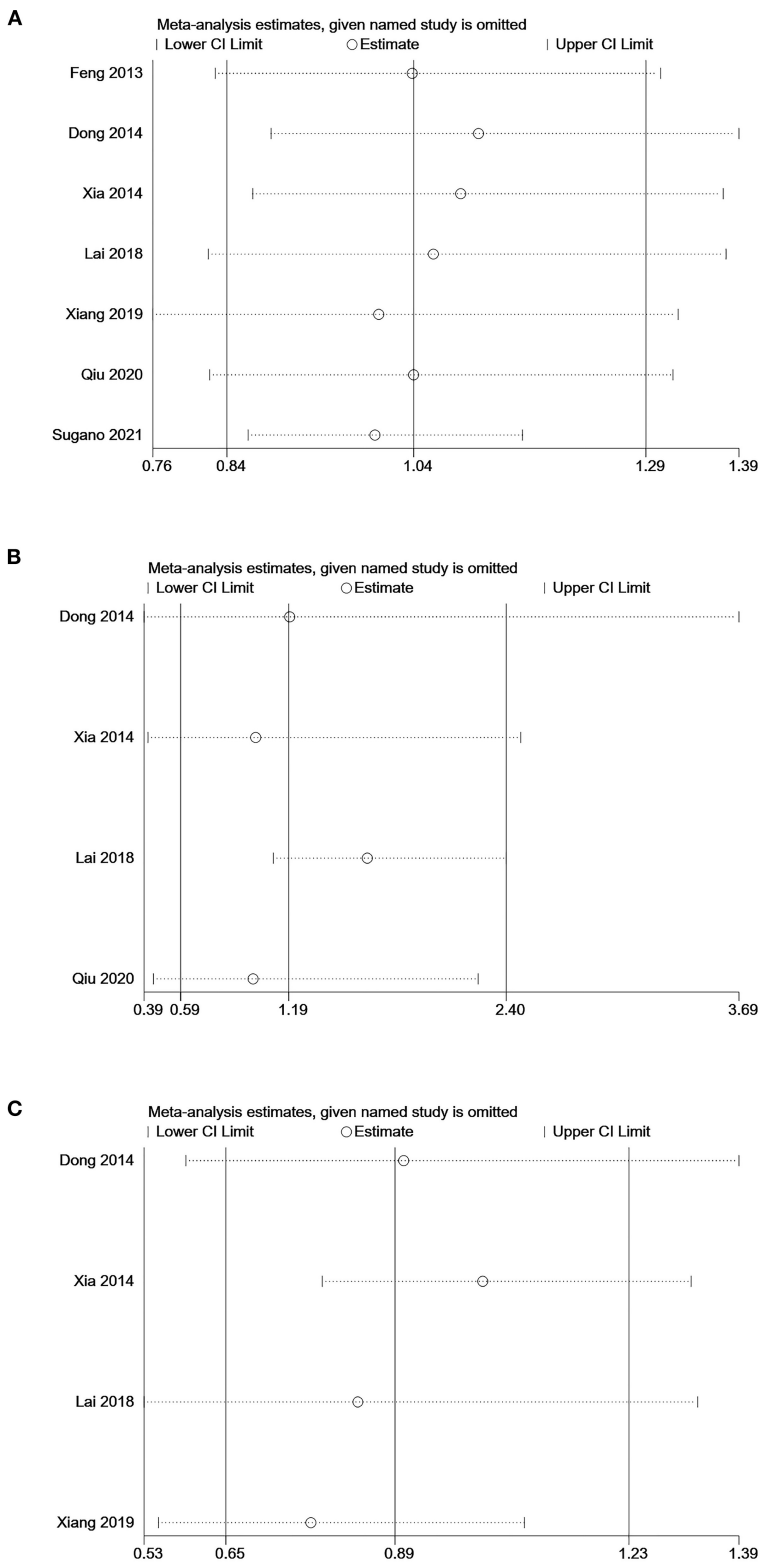
Sensitivity analysis of the association between serum uric acid levels and mortality in PD patients. **(A)** comparison: low vs. middle, outcome: all-cause mortality; **(B)** comparison: high vs. low, outcome: all-cause mortality; **(C)** comparison: low vs. middle, outcome: cardiovascular mortality.

Sensitivity analyses of the association between SUA levels (low vs. middle) and all-cause or cardiovascular mortality showed that omitting individual studies had no significant effect on the pooled results, suggesting good stability of the results (Figures 4A,C). Sensitivity analysis of the association between SUA levels (high vs. low) and all-cause mortality showed that the pooled result was significantly influenced by omitting Lai's study (21), indicating the stability of the result was relatively poor (Figure 4B).

### Subgroup Analysis

**Figure 5:**
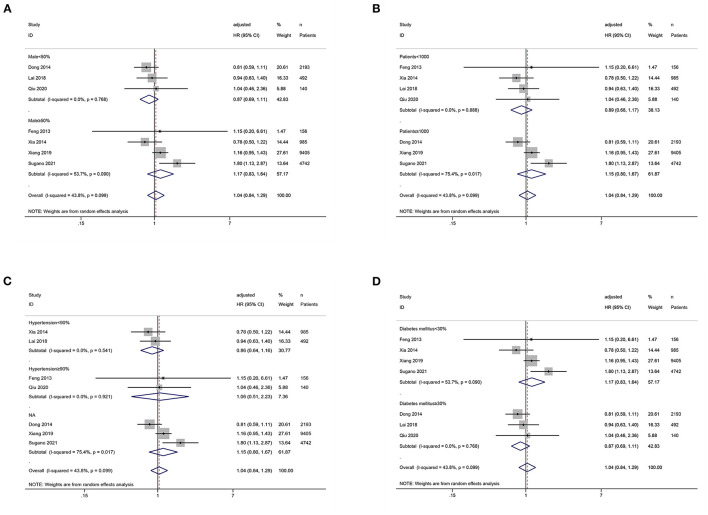
Subgroup analysis of the association between serum uric acid levels (low vs. middle) and all-cause mortality in PD patients. **(A)** by male proportion (50% as the cut-off value); **(B)** by sample size (1,000 as the cut-off value); **(C)** by hypertension proportion (90% as the cut-off value); **(D)** by diabetes mellitus proportion (30% as the cut-off value).

We conducted the subgroup analysis of the association between SUA levels (low vs. middle) and all-cause mortality. Subgroup analyses by male proportion (50% as the cut-off value), sample size (1,000 as the cut-off value), hypertension proportion (90% as the cut-off value), and diabetes mellitus proportion (30% as the cut-off value) were not statistically significant (*P* > 0.05) (Figures 5A–D).

### Meta-Regression Analysis

We used meta-regression to further explore the potential sources of heterogeneity, including male proportion (50% as the cut-off value), sample size (1,000 as the cut-off value), hypertension proportion (90% as the cut-off value), and diabetes mellitus proportion (30% as the cut-off value). Both univariate and multivariate analyses showed that the above factors were not the source of heterogeneity (*P* > 0.05) (Table 3).

**Table 3:** The results of meta-regression analysis.

**Covariates**	**Univariate analysis**			**Multivariate analysis**			
	**Exp(b)**	**95%*CI***	***P***	**Exp(b)**	**95%*CI***	***P***	**Adjusted *P***
Male (%)	1.31	0.75–2.30	0.264	1.28	0.53-3.09	0.442	0.703
Sample size	1.27	0.67–2.41	0.381	0.55	0.01-49.22	0.704	0.948
Hypertension (%)	1.09	0.87–1.37	0.352	1.32	0.27-6.38	0.613	0.863
Diabetes (%)	0.76	0.43–1.33	0.264	–	–	–	–

*Adjusted P was calculated by the Monte Carlo permutation test for meta-regression; Diabetes (%) was dropped in multivariate analysis because of collinearity*.

*CI, confidence interval*.

### Publication Bias

**Figure 6:**
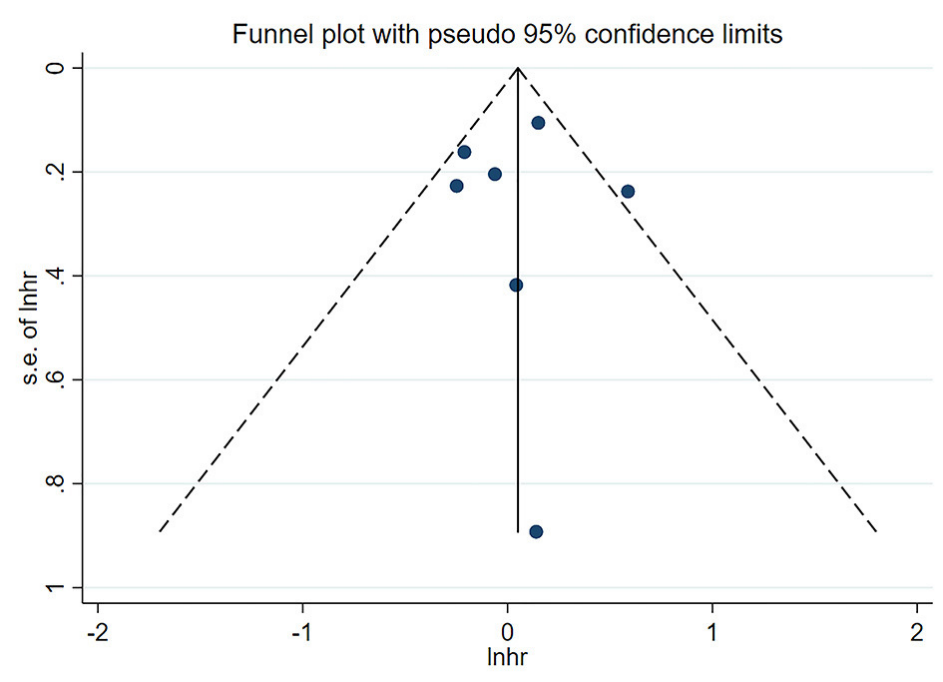
Funnel plot of the association between serum uric acid levels (low vs. middle) and all-cause mortality in PD patients.

Neither Begg's test nor Egger's test detected significant publication bias (*P* > 0.05) (Table 4). The shape of the funnel plot (low vs. middle, all-cause mortality) did not show visual evidence of asymmetry (Figure 6).

**Table 4:** Publication bias: Begg's test and Egger's test.

**Comparisons**		**Begg's test**		**Egger's test**	
		***Z***	***P***	***t***	***P***
All-cause mortality	Low vs. Middle	0.60	0.548	−0.11	0.915
	High vs. Middle	1.04	0.296	8.20	0.077
	High vs. Low	−0.34	1.000	0.00	0.998
Cardiovascular mortality	Low vs. Middle	1.70	0.089	−4.23	0.052
	High vs. Low	0.00	1.000	−0.35	0.785

*The level behind vs. is the reference group*.

### GRADE Framework for Evidence Quality

Based on the GRADE approach about prognosis (27), a body of observational evidence begins as high-quality evidence. The quality of evidence was moderate for all-cause mortality (low vs. middle; high vs. middle) and cardiovascular mortality (low vs. middle) due to inconsistency (different cut-off values for SUA classification). The quality of evidence for all-cause mortality (high vs. low) and cardiovascular mortality (high vs. low) was judged to be low due to the inconsistency (different cut-off values for SUA classification) and imprecision (wide 95% confidence interval) (Table 5).

**Table 5:** GRADE assessment of the quality of evidence.

**Comparisons**		**Risk of bias**	**Indirectness**	**Inconsistency**	**Imprecision**	**Publication bias**	**Rating up situations^a^**	**Quality**
All-cause mortality	Low vs. Middle	Not serious^b^	Not serious^c^	Serious^d^	Not serious^e^	Not serious^f^	None	Moderate
	High vs. Middle	Not serious^b^	Not serious^c^	Serious^d^	Not serious^e^	Not serious^f^	None	Moderate
	High vs. Low	Not serious^b^	Not serious^c^	Serious^d^	Serious^g^	Not serious^f^	None	Low
Cardiovascular mortality	Low vs. Middle	Not serious^b^	Not serious^c^	Serious^d^	Not serious^e^	Not serious^f^	None	Moderate
	High vs. Low	Not serious^b^	Not serious^c^	Serious^d^	Serious^g^	Not serious^f^	None	Low

*From Iorio et al. (27): in the field of prognosis, a body of observational evidence begins as high-quality evidence*.

a *Rating up situations included large effect, dose response gradient, and direction of plausible confounding*.

b *Risk of bias was judged low for individual studies (seven NOS scores or above) (see Table 2)*.

c *Appropriate population generalizability and outcomes applicability*.

d *Different cut-off values for SUA classification*.

e *Narrow 95% confidence interval*.

f *No publication bias detected (see Table 4)*.

g *Wide 95% confidence interval*.

## Discussion

To our knowledge, this is the first meta-analysis of the association between SUA levels and cardiovascular or all-cause mortality in PD patients. The pooled results showed that compared with middle SUA levels, high SUA levels were associated with increased all-cause mortality, while low SUA levels were not significantly associated with all-cause mortality. Besides, SUA levels might not be associated with cardiovascular mortality. The quality of evidence of this meta-analysis was judged to be moderate or low by the GRADE approach, which helps guide future research and clinical practice. Because current meta-analysis includes a relatively small number of studies, thus more large-sample and high-level studies are needed to determine the association between SUA levels and cardiovascular or all-cause mortality in PD patients. With the publication of relevant studies, this meta-analysis can be further updated in the future.

Our results showed that compared with middle SUA levels, high SUA levels were associated with an increased risk of all-cause mortality in PD patients. The possible reasons may be as follows: First, uric acid may induce oxidative stress by activating NADPH oxidase, stimulating the renin-angiotensin system (RAS), and disturbing mitochondrial function (33–35). Second, SUA can regulate inflammatory responses through a variety of cytokines (36). The pathophysiological mechanisms underlying the inflammatory state in CKD are complex and involve maladaptive cellular responses to injury, leading to sustained activation of proinflammatory and profibrotic signals (37, 38). Mouse models of CKD suggest that uric acid contributes to the pathophysiological processes described above. In the remnant kidney model, hyperuricemic rats exhibited higher blood pressure and proteinuria levels than normouricemic rats, which were histologically associated with greater glomerulosclerosis, interstitial fibrosis, and vascular lesions (39, 40). Third, hyperuricemia is associated with endothelial dysfunction. Uptake of uric acid into endothelial cells causes inflammation, oxidative stress, and eNOS dephosphorylation, leading to endothelial dysfunction by reducing NO bioavailability (41). Finally, high uric acid levels may reduce residual renal function in PD patients, leading to increased all-cause mortality (42, 43).

The pooled results indicated no significant association between SUA levels and cardiovascular mortality in PD patients, which might be due to the dual effects of SUA on the cardiovascular system. On the one hand, redundant SUA can cause inflammation, oxidative stress, endothelial dysfunction, and activation of the renin-angiotensin system, leading to cardiovascular disease (33–36, 41–45). On the other hand, many experiments have shown that uric acid is a potent free radical scavenger beneficial to the cardiovascular system (46, 47). Therefore, the association between SUA levels and cardiovascular mortality in PD patients may be balanced between protective and toxic effects. However, because only four related studies are included in this meta-analysis, and the individual study results are different, which may induce heterogeneity. Hence, the association between SUA levels and cardiovascular mortality in PD patients needs more high-level studies to validate.

Sensitivity analysis showed that compared with low SUA levels, high SUA levels were associated with an increased risk of all-cause mortality after excluding Lai's study (21). The results of Lai's study were significantly different from others, possibly because Lai's study had a longer median follow-up period (>3 years), while uric acid might have a more remarkable ability to predict mortality in PD patients within 3 years (32). Based on Lai's findings, the beneficial effect of UA on all-cause mortality seems to outweigh its harmful effect, and thus may have a protective effect in PD patients. This may be explained by the association between low SUA levels and malnutrition and may increase oxidative stress. Besides, different studies adjust for different confounders, the residual confounding bias may remain, which may contribute to the heterogeneity of results.

This meta-analysis has the following limitations: First, all the included studies were cohort studies, the potential confounders could not be adjusted entirely. Second, heterogeneity was observed among the included studies. Each study was adjusted for inconsistent confounding factors, with different cut-off values for SUA classification. Third, as a result of the limited number of relevant studies published, the number of studies included in this meta-analysis was insufficient and led to inadequate power to conduct subgroup and meta-regression analysis. Fourth, only few of the included studies reported SUA as a continuous variable and had different units, so we only considered SUA as a categorical variable.

## Conclusion

In conclusion, compared with middle SUA levels, high SUA levels are associated with an increased risk of all-cause mortality in PD patients. SUA levels may not be associated with cardiovascular mortality. More high-level studies, especially randomized controlled trials, are needed to determine the association between SUA levels and cardiovascular or all-cause mortality in PD patients.

## Data Availability Statement

Publicly available datasets were analyzed in this study. This data can be found at: PubMed, Embase, Web of Science, the Cochrane Library, CNKI, VIP, and Wanfang databases.

## Author Contributions

Z-qL, Z-wH, S-lK, FY, and Z-jH designed the study and drafted the manuscript. Z-qL, Z-wH, and S-lK extracted the data and evaluated the quality. C-cH, FC, and FH verified the data. Z-qL, Z-wH, and ZL analyzed the data. Z-qL, S-lK, FY, and Z-jH revised the manuscript. Z-qL, ZL, FY, and Z-jH interpreted the results. Z-qL, Z-jH, C-cH, FC, and FH incorporated comments for the co-authors and finalized the manuscript. All authors approved the final version of the paper.

## Funding

This study was supported by the key Clinical Specialty Discipline Construction Program of Fuzhou, Fujian, P.R.C, the Startup Fund for scientific research, Fujian Medical University (Grant number: 2019QH1297), and the Scientific Foundation of Fuzhou City (Grant number: 2020-WS-57).

## Conflict of Interest

The authors declare that the research was conducted in the absence of any commercial or financial relationships that could be construed as a potential conflict of interest.

## Publisher's Note

All claims expressed in this article are solely those of the authors and do not necessarily represent those of their affiliated organizations, or those of the publisher, the editors and the reviewers. Any product that may be evaluated in this article, or claim that may be made by its manufacturer, is not guaranteed or endorsed by the publisher.
